# Association of sexual function and psychological symptoms including depression, anxiety and stress in women with recurrent vulvovaginal candidiasis

**DOI:** 10.4274/jtgga.galenos.2019.2019.0077

**Published:** 2020-06-08

**Authors:** Zeinab Moshfeghy, Somayeh Tahari, Roksana Janghorban, Fatemeh Sadat Najib, Arash Mani, Mehrab Sayadi

**Affiliations:** 1 Student Research Committee, School of Nursing and Midwifery, Shahid Beheshti University of Medical Sciences, Tehran, Iran; 2 Student Research Committee, Department of Midwifery, School of Nursing and Midwifery, Shiraz University of Medical Sciences, Shiraz, Iran; 3 Department of Midwifery, School of Nursing and Midwifery, Community Based Psychiatric Care Research Center, Shiraz University of Medical Sciences, Shiraz, Iran; 4 Department of Obstetrics and Gynecology, School of Medicine, Infertility Research Center, Shiraz University of Medical Sciences, Shiraz, Iran; 5 Department of Psychiatry, School of Medicine, Community Based Psychiatric Care Research Center, Shiraz University of Medical Sciences, Shiraz, Iran; 6 Student Research Committee, Department of Biostatistics, Shiraz University of Medical Sciences, Shiraz, Iran

**Keywords:** Sexual dysfunction, stress, depression, anxiety, vulvovaginal candidiasis

## Abstract

**Objective:**

Recurrent vulvovaginal candidiasis (RVVC) is a common vaginal infection which could affect the quality of life, romantic relationships, and sexual performance. There is some evidence that psychological problems result in an increased incidence of RVVC by changing the immune systems of individuals. The aim of this study was to determine the association of sexual function and psychological factors including depression, anxiety, and stress in women with RVVC.

**Material and Methods:**

Study design was case controlled. Equal numbers of women with RVVC and uninfected women referred to gynecology clinics were selected, using convenience purposive sampling. Two samples of vaginal discharge were prepared from each person. One sample was examined microscopically and the second was cultured on Sabouraud Agar. Data collection tools used for this study included demographic questionnaire, Female Sexual Function Index, Depression Anxiety Stress Scales-21. Data were analyzed using SPSS software (version 19).

**Results:**

Less sexual satisfaction [odds ratio (OR): 0.608, 95% confidence interval (CI): 0.421-0.878] and less orgasm (OR: 0.741, 95% CI: 0.530-0.998) was associated with RVVC. In patients with RVVC, the levels of depression, anxiety and stress were significantly higher compared to those of healthy individuals.

**Conclusion:**

Depression, anxiety and stress in the past four weeks are related to an increased risk of RVVC. There is an association between depression, anxiety and stress, sexual satisfaction, and orgasm with RVVC. It may be that psychological interventions and sexual counseling can be effective in improving RVVC.

## Introduction

Genital tract infections are common problems in women (1,2). Vaginitis is an inflammation and infection of the vagina and its symptoms include itching or irritation, unusual and malodorous discharge, leukorrhea and dyspareunia (3). According to the World Health Organization candida, trichomonas, and bacterial infection are considered to be the main factors causing vaginitis and these three pathogens constitute approximately 90% of vaginal infections (1).

Candida albicans is responsible for 85-95% of vaginal yeast infections (4). This disease is mostly seen in women of reproductive age (5). Studies have shown that the occurrence of this problem is rare before the age of menarche and has been observed less commonly at postmenopausal ages (6,7). This indicates the existence of a hormonal dependency for the infection (8).

It is estimated that 75% of women will have a vaginal yeast infection at least once in their lifetime and around 45% of women will experience this type of infection two or more times (4). Causes of the vaginal yeast infections are various species of Candida, with Candida albicans being the most common cause accounting for 80-90% of the disease. Nevertheless, in patients with recurrent vaginal candidiasis, 15-47% of cases are caused by non-albicans species (9).

In studies carried out in different countries, the rate of infection with candidiasis is different. The rate in Nigeria was reported to be 6.5% and in Turkey, with culture method and clinical diagnosis, it has been reported as 17.4 and 14.1%, respectively (10,11). In a further study from Nigeria, the prevalence of this infection was reported as 18.9% (12). In Iran, in the cities of Tabriz, Sanandaj, Hamedan, Yazd, and Shiraz, the prevalence was reported to be between 25-45% (9). The occurrence of four episodes or more of candidiasis per year is called recurrent vulvovaginal candidiasis (RVVC) (4). The prevalence of RVVC, reported in a study from five European countries and the United States, was between 29% and 49% (13). In another study in Sweden in 2009, the incidence of RVVC, using fungal culture for diagnosis, was estimated as 29.8% (14). In Iran, in Sari, the incidence was reported as 24.2% (15).

Several factors play a role in the incidence of vaginal candidiasis, including antibiotics, pregnancy, diabetes mellitus, taking oral contraceptive pills, human immunodeficiency virus infection, wearing tight and nylon underwear with inadequate ventilation, vaginal douching, immunosuppression drugs, use of an intrauterine device, many sexual activities, local vaginal immune deficiency, using tampons instead of sanitary napkins, and oral sex. However, each of these factors could have an effect on incidence of recurrent chronic candida (4,5,6,16).

There is also some largely indirect evidence that psychological problems contribute to the incidence of RVVC (5). Depression, helplessness, hopelessness, and stressful life events may lead to the disease by inhibiting the immune systems of individuals. Studies have shown that there is a relationship between the central nervous system and the immune system, and consequently, the incidence of various stresses affects immune function and associated diseases (17,18,19). Chronic stress may reduce cellular immunity and affects the hypothalamic-pituitary-adrenal (HPA) axis reactions. If the HPA axis is active chronically, normal reaction to external acute stress may fail, and this will increase the susceptibility to inflammation and, crucially, infections (16,20).

In addition studies have shown that there was a significant relationship between sexual dysfunction in individuals with a diagnosis of depression, anxiety, and stress (21,22). Psychosocial factors, such as exposure to factors causing stress in daily life, can lead to disruption of the sexual response cycle (23). The results of a study from Brazil revealed that women with RVVC had lower scores in the domains of orgasm and sexual satisfaction in comparison to women with localized vulvar vestibulitis syndrome (24).

Therefore, the present study was conducted to investigate the possible influence of psychological factors on the incidence of RVVC, the role of the psychological condition on women’s sexual function and to address the lack of a study which could demonstrate an association between psychological factors (anxiety, stress, and depression) and sexual function in women with RVVC (5,16,25).

## Material and Methods

In this case control study, participants consisted of married Iranian women attending gynecology clinics and who had been sexually active in the past four weeks. The women were of reproductive age, and were able to complete the questionnaire or interview. Exclusion criteria included pregnant and lactating women, having a previous or current history of cancer, patients undergoing chemotherapy, having immune deficiency diseases, and being under treatment for sexual and/or psychological problems. In addition, women who were in their menstrual period, women who had intercourse in the past 24 hours, and those who used vaginal creams during the week prior to the study and used vaginal douching within 48 hours of the study were excluded, as were those not willing to participate in the study.

Fifty women with RVVC and 50 healthy women, referred to gynecology clinics were selected using convenience purposive sampling. From October 2015 to June 2016, the female researcher attended clinics every day. After explaining the objectives of the project and obtaining written informed consent from individuals who were willing to participate in the study, samples were obtained. After preliminary review, if a selected individual was not included in the study because of exclusion criteria, a further suitable recruit was obtained until the desired number of cases and of controls was achieved.

The participants in the case group were married women with a history of at least four episodes of vulvo-vaginal candidiasis per year. All cases had a documented diagnosis of symptomatic episodes of infection in their clinic records. The diagnosis was suggested clinically by gynecologists working at the recruitment clinics, according to the presence of external dysuria together with vulvar pruritus, pain, swelling, and/or redness and signs include vulvar edema, fissures, excoriations, and thick “curdy” vaginal discharge and microscope examination of a smear from the vagina using a potassium hydroxide mounting. Additionally, an experienced laboratory microbiologist gave a definitive diagnosis of recent candida infection using fungal cultivation and direct observation under a microscope slide. The control group consisted of healthy individuals who were referred to clinics for routine screening. After direct observation under a microscope slide preparation and taking samples of vaginal discharge culture, all of the controls were shown not to have RVVC.

The data were collected via three questionnaires. The first questionnaire included demographic characteristics, pregnancy and childbirth information, contraceptive methods, history of fungal infections, bacterial vaginosis and trichomonas, history of drug use, menstrual history, and each participants’ health information. The second questionnaire was the Female Sexual Function Index (FSFI) which was designed by Rosen et al. (26) and validated for use in an Iranian population by Mohammadi et al. (27). The FSFI is a 19-item questionnaire designed to determine the sexual function status in women in the last four weeks. It assesses six domains: Sexual desire (two questions); sexual excitation (four questions); lubrication (four questions); orgasm (three questions); satisfaction (three questions); and pain (three questions). The FSFI has 6 Likert options relating to sexual activity (never, rarely, sometimes, often, always), which are scored for each domain. Therefore, at least the total scale score is 2 and maximum has been considered as 36. Generally, higher score indicates better sexual function. According to Mohammadi et al. (27), reliability of scale and subscales for all the individuals was calculated as 0.85 for total score, 0.76 for sexual desire, 0.88 for sexual excitation, 0.88 for lubrication, 0.9 for orgasm, 0.71 for satisfaction and 0.87 for pain (27). The third questionnaire was the Depression Anxiety Stress Scales (DASS-21). The questionnaire, which is the short form, contains 21 questions and measures three domains of depression, anxiety, and stress of the individual in the four weeks prior to completing the questionnire. DASS-21 is scored on Likert scale. Each subscale of this questionnaire includes seven questions and the final score of each is obtained from the total score for each question. Each question is scored between zero (does not apply at all in my case) to 3 (completely true in my case). Given that this questionnaire is the shortened form of the original scale (42 questions), the final score for each of the subscales should be doubled (28). Lovibond and Lovibond reported a correlation of 0.54 between the two scales of depression and anxiety (29). The reliability and validity of the questionnaire was evaluated by Samani and Jokar (30) in Iran. The retest reliability for depression, anxiety, and stress scales was 0.80, 0.76 and 0.77, respectively, and Cronbach’s alpha for depression, anxiety, and stress was also reported as 0.81, 0.74 and 0.78, respectively (30).

After determining that the patients met the inclusion criteria, the demographic and the DASS-21 Questionnaire were completed by the participants during the first visit and the FSFI Questionnaire was completed during the second visit, when they were referred to clinics to know the culture results and drug prescription.

The participants included in the study in both groups were examined in the lithotomy position. By inserting a speculum, the researcher observed and assessed vaginal discharge in terms of color, odor, volume and other diagnostic features for Candida. Samples were collected from vaginal discharge and posterior fornix using a sterile cotton swab. Two swab samples were taken from each patient, one of which was mounted on a slide for microscopy. The second swab was sent for culture using Sabouraud’s dextrose agar under sterile conditions. Slides were marked with identifying information and, at the end of each working day, the slides and culture swabs were sent to the mycology laboratory of Faqihi Hospital for viewing under a microscope and culturing. Examination of samples was carried out by an expert mycologist. The expert added a drop of potassium hydroxide to discharges of the first slide for viewing under a microscope. In the second sample for culture, a swab was placed on Sabouraud Dextrose Agar. The detection of yeasts in culture samples was initially made by observing yeast groups, with false hyphae, under a microscope. This was then confirmed by standard culturing in the laboratory. Finally, women with positive microscopic results and positive swab cultures for fungi were assigned to the case group, while women with negative microscopic results and negative swab cultures for fungi were assigned to the control group.

The research was approved by the Ethics Committee of the Deputy of Research and Technology Shiraz University of Medical Sciences (approval number: 7592). Informed consent was obtained from all individual participants included in the study.

### Statistical analysis

Data were analyzed using SPSS software, version 19 (IBM Inc., Armonk, NY, USA). The following statistical tests were used, as appropriate: Independent t-test, chi-square test, logistic regression, and Pearson correlation. The significance level for all tests was 5%.

## Results

There was no significant relationship between demographic characteristics, method of contraception, or having specific dietary habits with recurrent Candida infection in both case and control groups. The history of infection in women with recurrent Candida infections (n=25/50) was significantly higher (χ^2^=7.25, p=0.001) than healthy women (n=12/50). There were no significant differences regarding the use of vaginal douching between the two groups. However, the case group individuals (n=30/50) were significantly more inclined (χ^2^=30.1, p<0.001) to wear tight clothes compared with the control group (n=4/50).

As shown in Table 1, the mean score of women’s sexual function in domains of orgasm (p=0.042), and satisfaction (p=0.005) was higher in the control group and this difference was statistically significant. However, in other domains, there were no statistically significant differences between the two groups. The overall sexual function values in the control group on average were a score of two greater than in the case group, and this was significant (p=0.043). In all domains, the mean of sexual function score in the case group was lower than in the control group.

When aspects of sexual function were investigated it was found that less sexual satisfaction [odds ratio (OR): 0.608, 95% confidence interval (CI): 0.421-0.878] and less orgasm (OR: 0.741, 95% CI: 0.530-0.998) were reported by women with a history of RVVC in the preceding four weeks. There were no significant differences between the two groups in the domains of desire, mental stimulation, lubrication, and pain (Table 2).

Frequency distribution of depression score (p<0.001), anxiety (p<0.001), and stress (p=0.037) in the two groups were compared using Fisher’s exact test or chi-square test. The results of this test showed the significance of the severity of these disorders in women with RVVC, such that the frequency distribution of these disorders in the case group compared to the control group showed a more severe situation.

Comparing mean scores for depression, anxiety, and stress in both groups showed that levels of these three measures of psychological function were significantly higher in the case group than the control group and the difference between the two groups was statistically significant (Table 3).

Self reported measures of depression, anxiety and stress in the past four weeks were associated with a history of RVVC (Table 4). In addition, a significant and inverse correlation was found between the domains of overall sexual function and depression, anxiety, and stress (Table 5).

## Discussion

The results showed that there were significant differences between the case and control groups regarding the overall sexual function score. Moreover, in the case group, sexual function score in all domains (desire, mental stimulation, lubrication, orgasm, satisfaction, and pain) was lower than the control group. However, in the domains of orgasm and satisfaction, this difference was statistically significant. This study showed that less sexual satisfaction was associated with a history of RVVC. However, the relationship of the other domains of sexual function with a history of RVVC was not significant.

In a previous study, the sexual function of 58 Brazilian women (11 patients with RVVC, 18 patients with localized vulvar vestibulitis and 29 healthy individuals) were investigated and it was found that both the individuals with RVVC and localized vestibulitis syndrome had significantly lower sexual function scores than the women in the control group. In addition, women with RVVC had significantly lower scores for satisfaction and orgasm domains (24). However, there was no significant difference found for other domains, which is consistent with the findings of the current research. Gungor et al. (31) in a study from Turkey, reported contrasting results. The study was conducted in 114 women in three groups. The first group included 58 women with no vaginal discharge, the second group included 29 women with abnormal vaginal discharge with itching and in the third group, 27 women had abnormal discharge without itching. Their results showed that women with abnormal vaginal discharge with or without itching had significantly higher overall score of sexual function compared to that of the control group. These differences may be attributed to subject selection bias, subjective reporting, possible cultural differences and differences in the diagnostic criteria for discharge culturing.

The results showed that there was a significant relationship between depression, anxiety, and stress and a history of RVVC. These findings suggest that levels of depression, anxiety, and stress in patients with RVVC are higher than healthy individuals. The result is consistent with another study which showed both chronic stress and reduced antioxidant capacity may be predisposing factors for RVVC. This implies that a dysregulation of immune function, which can be associated with poorer mental health parameters, may increase the risk of RVVC (16,20,32).

In a study the Short-Form Health Survey (SF-36) was used to measure health-related quality of life in 101 healthy women and 102 women with RVVC. The results showed that women with RVVC had lower physical and mental composite scores compared with controls (5). Although the scales used to measure stress and mental health in this and the current study were not similar, the results of both highlight that women with this infection report more stress. A further study showed that women with chronic vaginal symptoms such as RVVC, vestibulitis syndrome and inflammatory vulvovaginitis had high rates of mental disorders (33). Meyer et al. (34) suggested that psychosocial risk factors, particularly stress, were the main causes of RVVC.

The findings of the present study showed a significant and inverse correlation between the domains of sexual function and depression, anxiety, and stress. Mazinani et al. (35) showed that there was a significant relationship between psychiatric disorders, a history of psychiatric medicine and FSF. Although their study was not conducted on women suffering from RVVC, its findings regarding the significant relationship between mental disorders and sexual dysfunction are consistent with the present study. A study from Egypt showed that higher anxiety correlated with female sexual dysfunction (22), which again is consistent with the present study. Another study showed that trait anxiety and anxiety sensitivity were related to greater self-reported female sexual arousal outside the laboratory (36). The etiology of anxiety, not the experience of anxiety *per se*, seems to interfere adversely with sexual function.

Studies have shown a high prevalence of female sexual dysfunction in depressed women, regardless of type and severity of depression (25,37). Sexual dysfunction occurs at any stage of the sexual response cycle and reduces the quality of life of many women. Multiple psychological distresses could be sufficient evidence to suspect associated sexual problems (38).

It may be that a reduction in sexual satisfaction and orgasm can affect the mental state of women leading to an increase in stress, anxiety and depression. As these problems increase, they can affect the immune system and the body becomes more susceptible to infections, and ultimately, they lead to an increase in RVVC. As demonstrated by the results of this study, sexual function status in these women had an inverse association with mental disorders (anxiety, stress, and depression). Thus, if the status of sexual function has a lower score, these parameters of mental health are likely to be worse.

The present study has attempted to bridge the research gap in the field of mental health and sexual function in women with RVVC. This study should be considered as a preliminary study for the planning of larger, prospective interventional studies in patients with RVVC. This study has some limitations. The study was conducted only in Fars province. Additionally, due to the case-control design of study, we could not assess causal connection between mental disorders and RVVC. Cohort studies or randomized control trials with psychological intervention would be necessary to establish if poor mental health leads to an increased likelihood of RVVC and whether psychological intervention can aid in the recovery from this chronic infection.

## Conclusion

The results of the present study showed that the reduction in sexual satisfaction, orgasm and mental disturbances (anxiety, depression and stress) in the past month was associated with a history of RVVC. It has been suggested that poor mental health may be one of the causes of RVVC. In addition, this study showed an inverse relationship between sexual dysfunction and markers of mental health (stress, anxiety and depression) and has suggested that a reduction in sexual satisfaction and orgasm could increase anxiety, depression and stress, which may increase the likelihood of RVVC. Therefore, there may be a role for sexual counseling and psychotherapy techniques, such as relaxation, in order to enhance their mental performance, reduce stress and aid in the treatment of RVVC.

## Figures and Tables

**Table 1:**
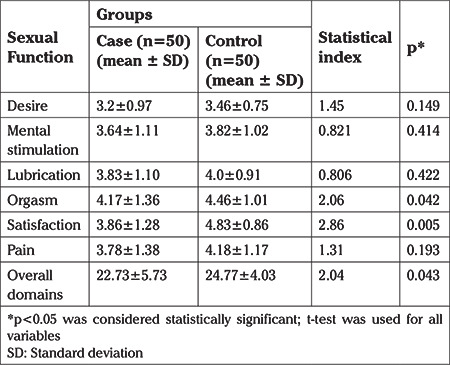
Comparison of mean score of sexual function in both case and control groups

**Table 2:**
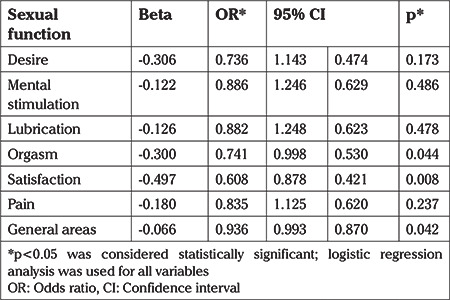
Association of sexual function with recurrent vulvovaginal candidiasis in both case and control groups

**Table 3:**
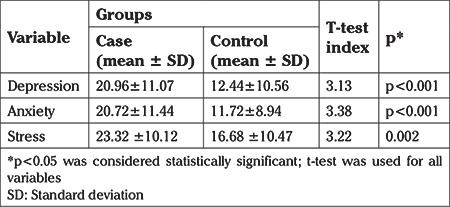
Comparison of mean scores of depression, anxiety and stress between the case and control groups

**Table 4:**
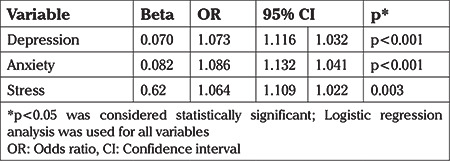
The association of depression, anxiety, and stress with recurrent vulvovaginal candidiasis in case and control groups

**Table 5:**
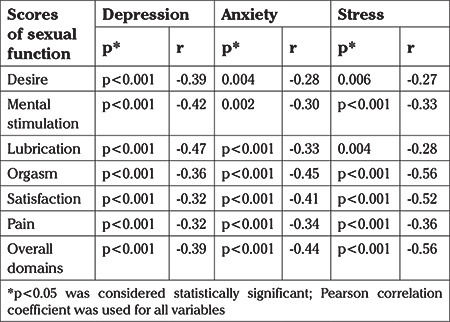
The correlation between the scores of different domains of sexual function and scores of depression, anxiety and stress in both case and control groups
